# Sepiapterin reductase gene‐disrupted mice suffer from hypertension with fluctuation and bradycardia

**DOI:** 10.14814/phy2.13196

**Published:** 2017-03-21

**Authors:** Chiho Sumi‐Ichinose, Yui Suganuma, Taiki Kano, Noriko Ihira, Hiroko Nomura, Kazuhisa Ikemoto, Tadayoshi Hata, Setsuko Katoh, Hiroshi Ichinose, Kazunao Kondo

**Affiliations:** ^1^ Department of PharmacologySchool of MedicineFujita Health UniversityToyoakeJapan; ^2^ Graduate School of Health ScienceFujita Health UniversityToyoakeJapan; ^3^ Department of DentistryMeikai UniversitySakadoJapan; ^4^ School of Life Science and TechnologyTokyo Institute of TechnologyYokohamaJapan

**Keywords:** Autonomic failure, endothelial dysfunction, hypertension, tetrahydrobiopterin

## Abstract

(6*R*)‐l‐*erythro*‐5,6,7,8‐Tetrahydrobiopterin (BH4) is an essential cofactor for monoamine and nitric oxide (NO) production. Sepiapterin reductase (SPR) catalyzes the final step in BH4 biosynthesis. We analyzed the cardiovascular function of adult *Spr* gene‐disrupted (*Spr* ^−/−^) mice for the first time. After weaning, *Spr* ^−/−^ mice suffered from hypertension with fluctuation and bradycardia, while the monoamine contents in these mice were less than 10% of those in the wild‐type mice as a result of BH4 depletion. Heart rate variability analysis indicated the sympathetic dominant state in *Spr* ^−/−^ mice. The endothelium‐dependent vascular relaxation in response to acetylcholine was significantly impaired in *Spr* ^−/−^ mice after sexual maturation (above 4 months old). Protein amounts of *α*_1_ adrenergic receptor and eNOS in the aorta were not altered. *Spr* ^−/−^ mice exhibited hypoglycemia and elevation of plasma renin activity. Our results suggest that the hypertension with fluctuation and bradycardia of *Spr* ^−/−^ mice would be caused by an imbalance of sympathetic and parasympathetic input and impaired nitric oxide production in endothelial cells. We suggest an important role of BH4 and SPR in age‐related hypertension and a possible relationship with the cardiovascular instabilities in autonomic diseases, including Parkinson's disease and spinal cord injury.

## Introduction

Sepiapterin reductase (SPR, EC 1.1.1.153) is the enzyme that catalyzes the final step in (6*R*)‐l‐*erythro*‐5,6,7,8‐tetrahydrobiopterin (BH4) biosynthesis. BH4 is an essential cofactor for aromatic amino acid hydroxylases, including tyrosine hydroxylase (TH), the rate‐limiting enzyme in catecholamine production (Thöny et al. 2000; Werner et al. 2011), and for all types of nitric oxide (NO) synthases (Kown et al. 1989; Tayeh and Marletta 1989). Studies on human *SPR* revealed an association with the *PARK3* locus in familial and sporadic Parkinson's disease (PD), although mutations in the SPR gene have not been identified (Sharma et al. 2006, 2011). *Spr* gene knockout (*Spr* ^−/−^) mice exhibit severe monoamine deficiencies caused by BH4 insufficiency and show phenotypes relating to dystonia/parkinsonism (Yang et al. 2006; Takazawa et al. 2008; Homma et al. 2013), probably due to dopamine deficiency in the brain (Blau et al. 2001; Friedman et al. 2012).

On the other hand, because norepinephrine is a main neurotransmitter for postganglionic sympathetic neurons, *Spr* ^−/−^ mice should exhibit cardiovascular dysfunction caused by severe deficiency of norepinephrine in the sympathetic nervous system and impairment of NO production. Forty percent of PD patients have orthostatic hypotension, which is often associated with supine or nocturnal hypertension (Sarabi and Goldstein 2011), and PD patients exhibit fluctuation of 100 mmHg in a day and a systolic blood pressure over 200 mmHg significantly more frequently than patients with other diseases (Tsukamoto et al. 2013). Patients suffering from multiple system atrophy (MSA) (Shannon et al. 2000; Fanciculli and Wenning 2015) and spinal cord injury (SCI) (Karlsson et al. 1998) also show unstable control of blood pressure, such as postprandial hypotension and reflective hypertension. However, the mechanisms underlying this fluctuation in blood pressure have not been clarified yet.

This is the first study to focus on the cardiovascular dysfunction of *Spr* ^−/−^ mice after sexual maturation, and it examined whether mutant mice mimic the sympathetic instabilities observed in patients with PD and several other conditions.

## Materials and Methods

### Experimental animals

*Spr* heterozygous (*Spr* ^+/−^) mice (OYC32) (Takazawa et al. 2008) were established by Lexicon Pharmaceuticals Inc. (Woodlands, TX.) and were crossed with C57BL/6JJcl mice for more than 10 generations. *Spr* ^+/−^ mice were crossed with BALB/cAJcl mice for four to six generations. We obtained homozygous *Spr* ^−/−^ mice (F1) by crossing BALB/cAJcl‐background *Spr* ^+/−^ female mice with *Spr* ^+/−^ C57BL/6JJcl‐background male mice. Genotypes were determined by PCR using the primer set of 5′GGAGGGTGTATCAGTGTCACTACGG3′ and 5′TGCCGGAAGTCAAACAGAGCATGGAG3′, which amplifies 335 bp of *Spr* wild‐type allele, and the primer set of 5′TCGTGCTTTACGGTATCGCCGCTCCCGATT3′ and 5′CTCCCCTACCCACCCACTCCTG3′, which amplifies 281 bp of mutated allele. We analyzed male F1 *Spr* ^−/−^ mice and their littermates as controls, except when determining blood pressure at the adult stage and in the electrocardiogram (ECG) study. The mice were kept in temperature‐ and humidity‐controlled conditions under a 12‐h light–dark cycle (lights on at 0800 h and off at 2000 h), with free access to normal chow and water. Mice were usually weaned at 1 month of age. All experiments and animal care were performed according to the guidelines for animal experiments of Fujita Health University and were approved by the committee of animal experiments (AP16043).

### Biochemical analysis

Two‐ to three‐ and four‐ to six‐month‐old male mice were anesthetized by intraperitoneal (i.p.) injection of pentobarbital (75 mg/kg), and total blood was collected in heparinized tubes from the axillary artery to prepare the plasma. Dissected tissues, the brain, liver, kidney, aorta, heart, lung, and adrenal glands were immediately frozen on dry ice and stored at −80°C until further analysis. Tissues were homogenized with buffer containing 50 mmol/L Tris‐HCl (pH 8.0), 100 mmol/L KCl, 0.1 mmol/L EDTA, 1 mmol/L dithiothreitol, 10% glycerol, 0.001 mg/mL pepstatin A, 0.002 mg/mL leupeptin, and 0.5 *μ*mol/L PMSF. After centrifugation at 15,000*g*, the supernatant was used for the assay. The protein concentration was quantified using the Bradford method (Bradford 1976). Homogenates were deproteinized by the addition of 0.2–0.05 mol/L perchloric acid. We quantified BH4 and dihydrobiopterin (BH2) separately using HPLC with fluorescence detection using a postcolumn oxidation method (Tani and Ohno 1993). BH4 and BH2 were separated by Inertsil ODS‐3 column (4.0 *μ*m, 3.0 mm × 250 mm, GL‐Science, Tokyo, Japan) using 0.1 mol/L Sodium phosphate buffer (pH 3.0) containing 5% methanol, 3 mmol/L Sodium 1‐octanesulfonate, 0.1 mmol/L EDTA, and 0.1 mmol/L ascorbic acid, and those were oxidized to biopterin by 20 mmol/L NaNO_2_, and then detected by fluorescence detection (Excitation; 375 nm, Emission 465 nm). Peak area was compared with that of biopterin standard. For determination of total biopterin, BH4, BH2, and quinonoid‐dihydrobiopterin, were oxidized with the addition of 1% I_2_ and 2% KI in 1 mol/L HCl for 1 h at room temperature (Fukushima and Nixon 1980). Biopterin was separated by Develosil C30‐UG‐5 column (5.0 *μ*m, 4.6 mm × 150 mm, Nomura Chemical, Seto, Japan) using 50 mmol/L ammonium phosphate buffer (pH 3.0) as a mobile phase, and quantified by fluorescence detection (Excitation; 350 nm, Emission 450 nm). The monoamine content was assayed using the HPLC‐electrochemical detection system as previously described (Sumi‐Ichinose et al. 2001). The contents of the metabolites of NO, nitrite, and nitrate, were determined using an NO_2_/NO_3_ assay fluorometric kit (Dojindo Lab, Mashiki) with plasma after ultrafiltration (Amicon Ultra‐0.5, Merck Millipore Co. Darmstadt, Germany). The plasma renin activity was determined using a SensoLyte 520 fluorometric mouse renin assay kit (AnaSpec. Inc., Fremont CA) and expressed as the concentration of renin, which was calculated from the molecular weight using the protein supplied as the standard. The plasma used in the determination of NO metabolites and renin activity was obtained from 4‐ to 6‐month‐old male mice.

### Histological analysis

Organs were dissected from deeply anesthetized 5‐month‐old male mice by intraperitoneal injection of pentobarbital (75 mg/kg) and fixed in 10% neutralized formalin followed by embedding. The paraffin blocks were sliced into 3‐*μ*m sections and stained using the hematoxylin–eosin method (the heart) or the Elastica van Gieson method (the aorta) (Muto Kagaku, Tokyo). Images were obtained using digital cameras on microscopes (SZX9 and IX73, Olympus, Tokyo). Two mice of each genotype were analyzed, and similar results were obtained.

### Determination of blood pressure and heart rate

Blood pressure and HR were determined using the tail‐cuff method (MK‐2000ST, Muromachi Kikai Co. Ltd., Tokyo) in conscious mice. To determine DBP, both SBP and mean blood pressure were measured for calculation. The measurements were generally performed between 0900 and 1200 h for 4 days (male) or 3 days (female). The appropriate mouse holders and tail‐cuff sensors were chosen according to the body weight. Generally, we used an S mouse holder and a C57 mouse NH sensor for mice whose body weights were 20–30 g, and we chose an SS mouse holder and a Neo40 NH sensor for mice whose body weights were 5–10 g. For the circadian and fluctuation study, SBP was measured at 1000, 1300, 1600, 1900, and 2200 h for 4 days using 4‐ to 6‐month‐old male mice. These measurements were made five times at each measurement for each mouse, and the mean ± SEM were calculated after excluding the highest and lowest values. The measurement at 2200 h was performed under red light. The fluctuation in SBP (ΔSBP) in a day was determined by calculating the difference between the average maximal SBP and average minimal SBP in an experimental day for each mouse, and ΔSBP at 1000 h was determined by calculating the difference between the average maximal SBP and average minimal SBP at 1000 h over four experimental days for each mouse. The HR was recorded simultaneously. The fluctuations in HR (ΔHR) were determined using the same method.

### ECG analysis

Six‐ to seven‐month‐old female *Spr* ^−/−^ mice and their littermates were anesthetized with intraperitoneal injections of pentobarbital (75 mg/kg), and experiments were performed in spontaneously breathing animals fixed in the supine position. CM5‐lead ECG was continuously recorded throughout the experiment at a 1000 Hz sampling rate using an MP150 biosignal recorder (BIOPAC Systems, Goleta, CA) with subcutaneously attached disposable electrodes. The R‐R interval was automatically recorded for the original electrocardiographic data for each 2‐min segment during normal sinus rhythm, which showed a stable baseline with no U wave, using AcqKnowledge Version 3.9 (Biopac Systems. Inc.). The starting point of the Q waves and the end point of the T waves were automatically determined by numerical derivation of the entire ECG wave. Frequency analysis for the measurement of heart rate variability (HRV) was performed at intervals of 120 sec. Power spectra for HRV were obtained using the fast Fourier transformation (FFT) autocorrelation method. Low‐frequency (LF, 0.1–1.5 Hz), high‐frequency (HF, 1.5–5.0 Hz), and total frequency (TF, 0.0‐5.0 Hz) components were calculated from the HRV power spectra, as previously defined for the mouse species (Rajendra et al. 2006; Thireau et al. 2008).

### Determination of vascular tension

One‐ to two‐millimeter‐long ring segments were dissected from the upper side of the thoracic aorta of 2‐ to 3‐ and 4‐ to 8‐monthold male mice. The segments were mounted onto a DMT 610M multiwire myograph system (Danish Myo Technology, Aarhus, Denmark). The transmural pressure was set at 13.3 kPa at the beginning of each experiment by normalization program based on Mulvany and Halpern (1977) to standardize the condition of different vascular segments. The buffer was continuously bubbled with 95% O_2_ and 5% CO_2_ and maintained at 37°C, and 10^−5^ mol/L indomethacin was added to inhibit the synthesis of prostanoids. Aortic rings were precontracted several times in KPSS buffer (123.7 mmol/L KCl, 1.17 mmol/L MgSO_4_, 1.18 mmol/L KH_2_PO_4_, 2.5 mmol/L CaCl_2_, 25 mmol/L NaHCO_3_, 0.03 mmol/L EDTA, and 5.50 mmol/L glucose). For the first experiment, the aortic rings were contracted by the cumulative addition of phenylephrine, an *α*_1_‐selective agonist (10^−7^ mol/L to 3 × 10^−4^ mol/L) in PSS buffer (118.99 mmol/L NaCl, 4.69 mmol/L KCl, 1.17 mmol/L MgSO_4_, 1.18 mmol/L KH_2_PO_4_, 2.50 mmol/L CaCl_2_, 25 mmol/L NaHCO_3_, 0.03 mmol/L EDTA, and 5.50 mmol/L glucose). Contraction was indicated as the percentage relative to that obtained by 123.7 mmol/L KCl. In the second experiment, aortic rings submaximally precontracted by the addition of phenylephrine (usually 3 × 10^−7^ mol/L) were relaxed by the cumulative addition of acetylcholine (ACh) (10^−9^ mol/L to 3 × 10^−4^ mol/L). In the third experiment, vascular segments precontracted by phenylephrine were relaxed by the addition of ACh in the presence of 10^−4^ mol/L NG‐nitro‐l‐arginine methyl ester (l‐NAME), a nitric oxide synthase (NOS) inhibitor. In the fourth experiment, the precontracted aortic rings were relaxed by the cumulative addition of sodium nitroprusside (SNP), an NO donor (10^−10^–10^−6^ mol/L) in the presence of l‐NAME. Relaxation resulting from the addition of ACh or SNP was expressed as the percentage of the precontracted vascular tension induced by phenylephrine.

### Western blotting

Dissected aortas were homogenized with buffer containing 50 mmol/L Tris‐HCl (pH7.4), 150 mmol/L NaCl, 1% Nonidet P‐40, 1 mmol/L EDTA, 1% protease inhibitor cocktail (P8340) (Sigma‐Aldrich, Darmstadt, Germany), and 1% phosphatase inhibitor cocktail (167‐24381) (Wako Pure Chemical Industries, Osaka, Japan). The homogenate containing 100 *μ*g (for *α*_1_ adrenergic receptor) or 50 *μ*g protein (for eNOS) was separated by 6% SDS‐polyacrylamide gel electrophoresis, and transblotted to PDVF membrane (Bio‐Rad, Hercules). *α*_1_ Adrenergic receptor was detected by ab3462 (Abcam, Cambridge, UK) and eNOS was detected by 610296 (BD Science, Franklin Lakes) as a primary antibody, respectively. The membranes were striped and reincubated with anti‐*β*‐actin antibody (AC‐15) (Sigma‐Aldrich, St Louis) for normalization. Signals were detected by horse radish peroxidase‐conjugated secondary antibodies and ECL select (GE Healthcare Life Science, Buckinghamshire, UK). The density of the signals was then quantified by LAS 4000mini using ImageQuant TL software (GE Healthcare Life Science Buckinghamshire, UK).

### Determination of blood glucose

Serum was collected from pentobarbital‐anesthetized (75 mg/kg, i.p.) 4‐ to 6‐month‐old male mice after 17 h of fasting and analyzed with a Hitachi 7180 Auto Analyzer using the HK‐G6PDH method (Oriental Kobo Inc., Nagahama).

### Feeding behavior and blood pressure

Four‐ to six‐month‐old male mice were starved for 17 h, and SBP and HR were determined using the tail‐cuff method at 1000 h. The mice were then fed pellets. One hour after feeding, SBP and HR were determined again using the same method. The fluctuation in SBP and HR of each mouse before and after feeding was compared.

### Statistics

The values were expressed as the average ± SEM. Two‐way ANOVA, one‐way ANOVA, and two‐way ANOVA with repeated measurements followed by the Tukey–Kramer test as the post hoc comparison, the Χ^2^ test, Student's *t*‐test, and the Mann–Whitney U nonparametric test were applied.

## Results

### Survival ratio of Spr^−/−^ mice on a BALB/c‐C57BL/6 mixed background

*Spr* ^−/−^ mice on the mixed background of BALB/c‐C57BL/6 showed poor body weight gain, as previously reported (Homma et al. 2011, 2013); however, 79.6% of the weaned *Spr* ^−/−^ mice reached 4 months (male; 76.7%, female; 84.2%). There was no significant difference between the survival ratios of the two genders (*P* = 0.52, *n* = 30 for male, 19 for female, Χ^2^ test).

### BH4 and monoamine were depleted in the tissues of Spr^−/−^ mice

We performed biochemical analysis of the brains and other peripheral tissues from *Spr* ^−/−^ mice and their wild‐type littermates as controls to verify the BH4 and catecholamine deficiency in the adults using 2‐ to 3‐month‐old mice (Table 1). The body weight of *Spr* ^−/−^ mice (8.8 ± 1.0 g) was significantly lower than that of wild‐type controls (28.4 ± 0.8 g) (*P* < 0.01, *n* = 6, one‐way ANOVA with Tukey–Kramer as a post hoc). The BH4 and BH2 content in the brains of the *Spr* ^−/−^ mice was significantly lower than that in the wild‐type animals, and the norepinephrine and dopamine content was severely depleted. The serotonin content in the brains of *Spr* ^−/−^ mice (1.50 ± 0.26 pmol/mg protein) was also reduced to 8.6% of that in the wild‐type mice (17.4 ± 1.51 pmol/mg protein) (*P* < 0.01, *n* = 5, Student's *t*‐test). These results were consistent with the findings obtained in previous reports about *Spr* ^−/−^ mice at 4 weeks or 17 days old (Yang et al. 2006; Takazawa et al. 2008). Depletion of BH4 and monoamines was also confirmed in the peripheral tissues of *Spr* ^−/−^ mice. In the aortas of the wild‐type mice, the BH4 and BH2 contents were 1.24 ± 0.11 pmol/mg protein and 0.22 ± 0.06 pmol/mg protein, respectively, and the norepinephrine content was 6.85 ± 1.57 pmol/mg protein. The content of BH4 and BH2 in the aortas of the *Spr* ^−/−^ mice was below the detection limit, and norepinephrine content was 0.59 ± 0.26 pmol/mg, which was 8.6% of that in wild‐type mice. The BH2/BH4 ratios were 0.07–0.23 in the tissues of wild‐type mice, and those in *Spr* ^−/−^ mice were 0.03–0.29. We also determined the biopterin and monoamine content in the tissues of *Spr* ^−/−^ mice and wild‐type controls at 4–6 months old and obtained similar results (Table 2). The serotonin content in the brain of *Spr* ^−/−^ mice (1.04 ± 0.11 pmol/mg protein) was also reduced to 7.3% of the wild‐type (14.2 ± 1.11 pmol/mg protein) at this age (*P* < 0.01, *n* = 6, Student's *t*‐test). Because most of the total biopterin content is generally present in the tetrahydro form in tissues, the reduction in biopterin content obtained in this study reflects the reduction in BH4 (Fukushima and Nixon 1980; Takazawa et al. 2008).

**Table 1:** Biochemical analysis of 2‐ to 3‐month‐old wild‐type mice and *Spr* ^−/−^ mice

(A) BH4, BH2 and catecholamine content in the brain and peripheral tissue						
	Brain	Liver	Kidney	Aorta	Heart	Lung
	(*pmol/mg protein*)					
BH4						
*Spr* ^+/+^	5.44 ± 0.19	20.1 ± 1.2	3.56 ± 0.20	1.24 ± 0.11	0.96 ± 0.18	3.94 ± 0.33
*Spr* ^−/−^	1.93 ± 0.06^b^	2.38 ± 0.37^b^	1.17 ± 0.25^b^	–	0.35 ± 0.03^b^	0.18 ± 0.04^b^
	(35.5)	(11.6)	(32.9)		(36.5)	(4.6)
BH2						
*Spr* ^+/+^	0.36 ± 0.06	2.35 ± 0.42	0.34 ± 0.04	0.22 ± 0.06^b^	0.22 ± 0.02	0.40 ± 0.02
*Spr* ^−/−^	0.14 ± 0.00^a^	0.69 ± 0.05^b^	0.04 ± 0.01^b^	–	0.04 ± 0.01^b^	–
	(38.9)	(29.4)	(10.8)		(18.2)	
Norepinephrine						
*Spr* ^+/+^	14.6 ± 0.78	nd	3.94 ± 0.14	6.85 ± 1.57	9.62 ± 1.23	nd
*Spr* ^−/−^	0.83 ± 0.08^b^	nd	–	0.59 ± 0.26^b^	0.21 ± 0.05^b^	nd
	(5.7)			(8.6)	(2.2)	
Dopamine						
*Spr* ^+/+^	45.2 ± 0.99	nd	1.03 ± 0.09	–	0.91 ± 0.10	nd
*Spr* ^−/−^	1.45 ± 0.16^b^	nd	0.48 ± 0.04^b^	–	–	nd
	(3.2)		(46.6)			

(B) BH4, BH2 and catecholamine content in the adrenal gland				
	BH4	BH2	Epinephrine	Norepinephrine
	(*pmol/mg protein*)		(*nmol/mg protein*)	
*Spr* ^+/+^	24.5 ± 1.3	3.90 ± 0.42	15.2 ± 1.44	6.98 ± 0.53
*Spr* ^−/−^	0.96 ± 0.12^b^	–	0.59 ± 0.10^b^	0.39 ± 0.05^b^
	(3.9)		(3.9)	(5.6)

Percentage of the value of *Spr*−/− mice to those of wild‐type ones are indicated in the parentheses. Student's *t*‐test (*n* = 3–5). nd, not determined. –, under the detection limit.

a *P* < 0.05

b *P* < 0.01

**Table 2:** Biochemical analysis of 4‐ to 6‐month‐old wild‐type mice and *Spr* ^−/−^ mice

(A) Biopterin and catecholamine content in the brain and peripheral tissue						
	Brain	Liver	Kidney	Aorta	Heart	Lung
	(*pmol/mg protein*)					
Biopterin						
*Spr* ^+/+^	3.89 ± 0.17	12.7 ± 0.68	6.07 ± 0.43	0.86 ± 0.25	1.74 ± 0.24	5.35 ± 0.55
*Spr* ^−/−^	1.78 ± 0.12^a^	0.25 ± 0.02^a^	0.97 ± 0.16^a^	–	0.67 ± 0.03^a^	0.51 ± 0.08^a^
	(46)	(2.0)	(16)		(39)	(9.5)
Norepinephrine						
*Spr* ^+/+^	9.97 ± 0.72	nd	5.88 ± 0.46	8.37 ± 1.24	23.9 ± 2.30	13.3 ± 3.07
*Spr* ^−/−^	0.51 ± 0.05^a^	nd	–	–	0.45 ± 0.07^a^	–
	(5.1)				(1.9)	
Dopamine						
*Spr* ^+/+^	29.1 ± 2.24	nd	0.69 ± 0.16	–	1.96 ± 0.59	nd
*Spr* ^−/−^	1.08 ± 0.26^a^	nd	–	–	–	nd
	(3.7)					

(B) Biopterin and catecholamine content in the adrenal gland			
	Biopterin	Epinephrine	Norepinephrine
	(*pmol/mg protein*)	(*nmol/mg protein*)	
*Spr* ^+/+^	24.9 ± 2.36	19.6 ± 2.66	8.45 ± 1.14
*Spr* ^−/−^	1.38 ± 0.13^a^	0.92 ± 0.09^a^	0.36 ± 0.03^a^
	(5.5)	(4.7)	(4.3)

Percentage of the value of *Spr*−/− mice to those of wild‐type ones are indicated in the parentheses. *n* = 4–6. nd, not determined; –, under the detection limit.

a *P* < 0.01.

### Spr^−/−^ mice exhibited hypertension and bradycardia after weaning

**Figure 1:**
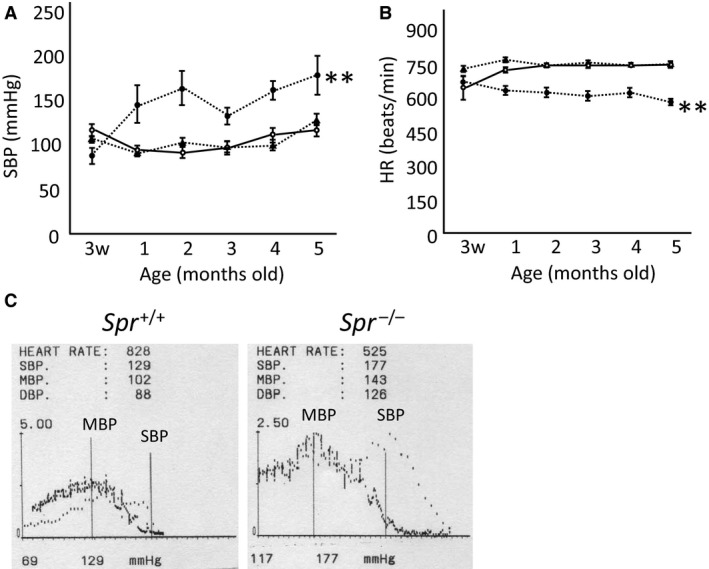
Developmental profile of *Spr* ^−/−^ mice, which suffered from hypertension and bradycardia after weaning. (A) Systolic blood pressure (SBP). (B) Heart rates (HR). The data from wild‐type (open circles with solid line), *Spr* ^+/−^ (solid triangles with dashed line), and *Spr* ^−/−^ (solid circles with dashed line) mice between 3 weeks old and 5 months old are indicated. Significant differences between *Spr* ^−/−^ mice and mice of the other two genotypes are shown with asterisks (**;*P* < 0.01, *n* = 5 and 6, two‐way ANOVA with repeated measurement with the Tukey–Kramer test as a post hoc). (C) Exemplary traces of the blood pressure recordings of a *Spr* ^+/+^ mouse (left panel), and a *Spr* ^−/−^ mouse (right panel). Vertical axis indicates the intensity of pulse wave (arbitrary unit), and horizontal axis indicates the pressure of the cuff (mmHg). SBP is determined as the detection point of the pulse wave, and MBP (mean blood pressure) is determined by the maximal point of the pulse wave by photoplethysmography, and those are indicated by bars. SBP and the intersection of the two axes (SBP‐60 mmHg), which is automatically set by the recorder, are printed below the horizontal axis.

**Figure 2:**
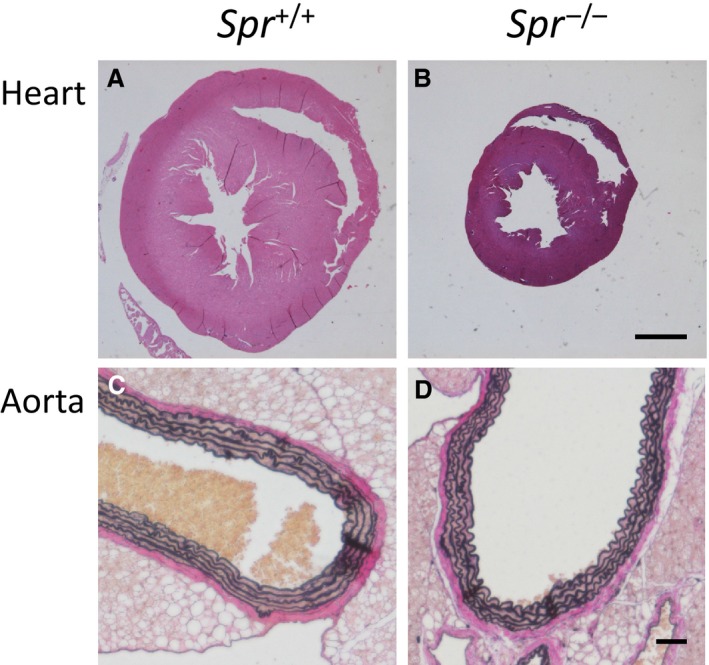
Histological study of the heart and aorta in *Spr* ^−/−^ mice and wild‐type mice. (A) The heart of wild‐type mouse. (B) The heart of *Spr* ^−/−^ mouse. (C) The aorta of wild‐type mouse. (D) The aorta of *Spr* ^−/−^ mouse. The hearts (A and B) were stained using the hematoxylin‐eosin method. The aortas (C and D) were stained using the Elastica van Gieson method. The scale bar in panel B indicates 1 mm. The scale bar in panel D indicates 50 *μ*m.

We determined the SBP and HR of male *Spr* ^−/−^ mice of 3 weeks to 5 months old, and it revealed that the hypertension and bradycardia of the *Spr* ^−/−^ mice became evident after weaning, at 1 month old, while, there were no significant differences between the SBP and the HR of the wild‐type mice and those of the heterozygous *Spr* ^+/−^ mice (Fig. 1A and B). At 4–6 months old, the SBP of the *Spr* ^−/−^ mice was significantly higher than that of the wild‐type mice of both genders (Table 3). The DBP of male *Spr* ^−/−^ mice was also significantly higher than that of wild‐type mice. In contrast, the HR of *Spr* ^−/−^ mice was significantly lower than that of wild‐type animals of both genders. The heart/body index of male *Spr* ^−/−^ mice was significantly higher than that of wild‐type animals; however, histological investigation revealed that there was not obvious hypertrophy of the left ventricles in the hearts or the tunica media in the aortas of the *Spr* ^−/−^ mice (Fig. 2).

**Table 3:** Body weight, blood pressure, heart rate, and heart/body weight index of 4‐ to 6‐month‐old wild‐type mice and *Spr* ^−/−^ mice

	Male		Female	
	*Spr* ^+/+^	*Spr* ^−/−^	*Spr* ^+/+^	*Spr* ^−/−^
Body weight (g)	41.8 ± 1.5	9.9 ± 0.5^a^	28.5 ± 0.7^c^	9.1 ± 0.6^a^
Systolic blood pressure (mmHg)	114.9 ± 6.7	174.6 ± 15.9^a^	117.5 ± 5.7	155.9 ± 16.7^b^
Diastolic blood pressure (mmHg)	61.3 ± 4.3	105.3 ± 15.6^b^	67.6 ± 6.1	90.8 ± 14.4
Heart rate (beats/min)	682.9 ± 15.5	565.5 ± 13.4^a^	684.7 ± 11.7	590.8 ± 11.8^a^
Heart/body weight index	0.44 ± 0.02	0.60 ± 0.02^a^	nd	nd

Two‐way ANOVA with the Tukey–Kramer as a post hoc comparison was performed for body weight, systolic blood pressure, diastolic blood pressure, and heart rate. One‐way ANOVA with the Tukey–Kramer as a post hoc comparison was performed for heart/body index. nd, not determined.

a *P* < 0.01 between *Spr* ^+/+^ and *Spr* ^−/−^ within each gender,

b *P* < 0.05 between *Spr* ^+/+^ and *Spr* ^−/−^ within each gender,

c *P* < 0.01 between male mice and female ones of *Spr* ^+/+^, *n* = 6.

### Fluctuation in the blood pressure of Spr^−/−^ mice

**Figure 3:**
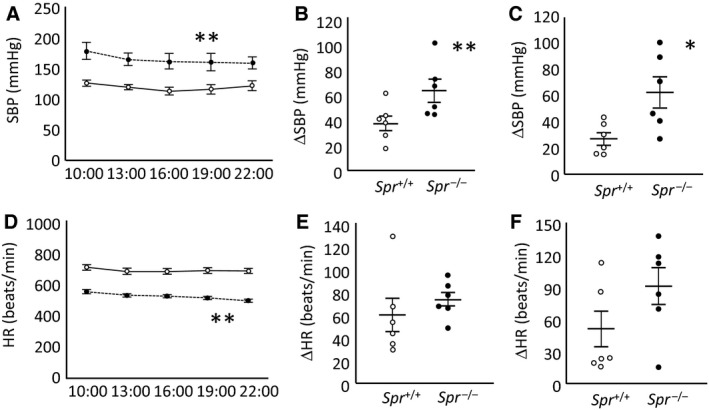
Measurement of systolic blood pressure and heart rate for 12‐hours; *Spr* ^−/−^ mice exhibited hypertension with fluctuation. (A) The systolic blood pressure (SBP). (D) Heart rate (HR). Those data were obtained from measurement at 1000, 1300, 1600, 1900, and 2200 h. Wild‐type mice; open circles with solid line, *Spr* ^−/−^ mice; solid circles with dashed line. The data are presented as the average ± SEM. The two genotypes in panels A and D are significantly different (**;*P* < 0.01, *n* = 6, two‐way ANOVA with repeated measurement with the Tukey–Kramer as a post hoc). (B) The fluctuation of SBP (ΔSBP) in a day per mouse. (E) The fluctuation of HR (ΔHR) in a day per mouse. (C) ΔSBP at 1000 h over 4 days per mouse. (F) ΔHR at 1000 h over 4 days per mouse. Each circle in panels of B, C, E, and F represents one mouse sample. Wild‐type mice; open circles, *Spr* ^−/−^ mice; solid circles. Significant differences between the two genotypes are indicated with asterisks (**;*P* < 0.01, *; *P* < 0.05, *n* = 6, Student's *t*‐test).

Next, we measured the SBP and HR of male *Spr* ^−/−^mice during a 3‐hour interval between 1000 and 2200 h to assess the circadian rhythms and compared the measurements with those from wild‐type animals. The average SBP of *Spr* ^−/−^ mice was consistently significantly higher than that of wild‐type mice (Fig. 3A). However, interestingly the ΔSBP of each *Spr* ^−/−^ mouse in a day (63.5 ± 9.0 mmHg) was significantly wider than that in wild‐type mice (37.7 ± 6.3 mmHg) (Fig. 3B), and the fluctuation at 1000 h over 4 days of each *Spr* ^−/−^ mouse (62.8 ± 12.3 mmHg) was also significantly wider than that in wild‐type mice (27.2 ± 5.1 mmHg) (Fig. 3C). The simultaneously recorded HRs of *Spr* ^−/−^ mice were significantly and constantly lower than those of wild‐type mice (Fig. 3D); however, ΔHR of each mouse in a day (Fig. 3E) and ΔHR at 1000 h of each mouse over 4 days (Fig. 3F) were not significantly different between the two genotypes (*P* = 0.41 and 0.13, respectively).

### HRV analysis of Spr^−/−^ mice suggested a sympathetic dominant state

Next, we recorded the ECGs of mice under pentobarbital anesthesia and assessed the function of the autonomic nervous system using R‐R interval‐spectral analysis. The ratios of LF/HF (3.175 ± 0.712) and LF/TF (0.349 ± 0.045) were significantly higher in the *Spr* ^−/−^ mice than the wild‐type animals (0.595 ± 0.268 and 0.075 ± 0.024, respectively) (*P* < 0.01, *n* = 5, Mann–Whitney U nonparametric test). These results suggest that the HRV parameters reflecting the sympathovagal balance sharply increased in *Spr* ^−/−^ mice in the sympathetic dominant state.

### Endothelium‐dependent relaxation was impaired in the aortas of Spr^−/−^ mice after sexual maturation

**Figure 4:**
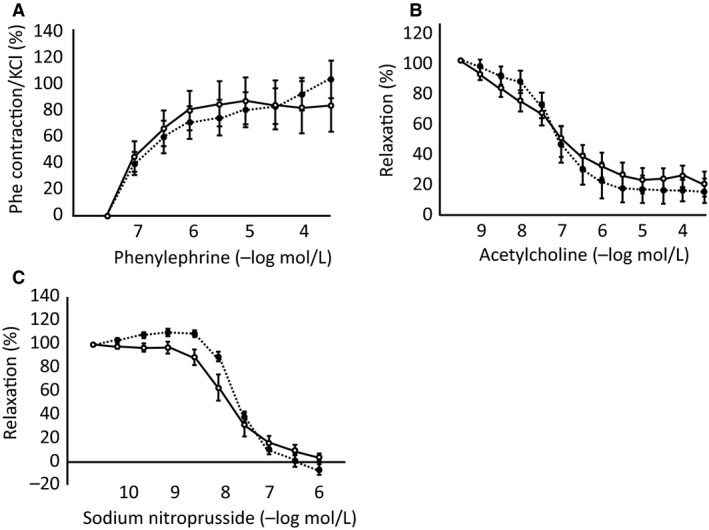
Vascular response curve of aortic rings isolated from 2‐ to 3‐month‐old wild‐type mice and *Spr* ^−/−^ mice. (A) Vasocontraction in response to phenylephrine relative to high KCl. (B) The endothelium‐dependent vascular relaxation response to acetylcholine (ACh). (C) The response to sodium nitroprusside (SNP) in the presence of L‐NAME. Wild‐type mice; open circles with solid line, *Spr* ^−/−^ mice; closed circles with dashed line. Differences in A, B, and C are not significant (*n* = 6, two‐way ANOVA with repeated measurement).

**Figure 5:**
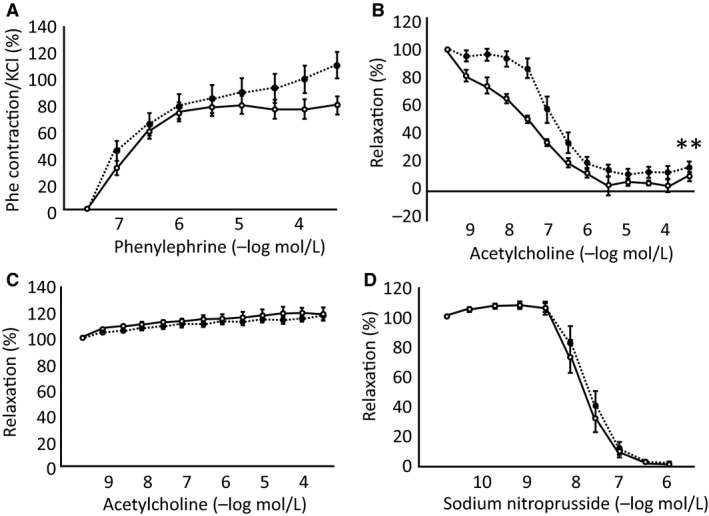
Vascular response curve of aortic rings isolated from 4‐ to 8‐month‐old wild‐type mice and *Spr* ^−/−^ mice. (A) Vasocontraction in response to phenylephrine relative to high KCl. (B) The endothelium‐dependent vascular relaxation response to acetylcholine (ACh). The vasodilatory response was significantly impaired in the aortic rings dissected from *Spr* ^−/−^ mice compared to those from their wild‐type littermates. (C) The vasodilatory response of the aortic segments from the *Spr* ^−/−^ mice and their wild‐type littermates to ACh in the presence of L‐NAME. The relaxation response to ACh disappeared in both genotypes. (D) The vasodilatory response to sodium nitroprusside (SNP) in the presence of L‐NAME. Wild‐type mice; open circles, *Spr* ^−/−^ mice; closed circles. Significant differences between the two genotypes are indicated with asterisks in B (**;*P* < 0.01, *n* = 10, two‐way ANOVA with repeated measurement with the Tukey–Kramer test as a post hoc). There was no significant difference between the aortic segments from *Spr* ^−/−^ mice and their wild‐type littermates in A, C and D.

To understand the mechanism of hypertension, we analyzed vascular function using aortic ring segments from *Spr* ^−/−^ mice and compared the results with those from wild‐type mice at two separate ages of 2–3 months (young adult) and 4–8 months. The young adult *Spr* ^−/−^ mice (2–3 months old) showed significantly higher SBP (136.4 ± 7.8 mmHg) and lower HR (609.3 ± 16.5 beats/min) than wild‐type animals in the same age (105.0 ± 5.4 mmHg and 677.9 ± 15.7 beats/min, respectively) (*P* < 0.01; SBP, *P* < 0.05; HR, *n* = 6, one ‐way ANOVA with Tukey–Kramer as a post hoc). The contraction of the aortic rings from the young adult *Spr* ^−/−^ mice in response to high KCl (123.7 mmol/L) (1.60 ± 0.48 mN) was not significantly different from that in wild‐type mice (1.09 ± 0.27 mN) (*P* = 0.37, *n* = 6, Student's *t*‐test). The contractile response to phenylephrine relative to high KCl and the vasodilatory responses to ACh and SNP in the presence of l‐NAME of the aortic rings from the young adult *Spr* ^−/−^ mice were not significantly different from those in wild‐type mice (*P* = 0.95, 0.73 and 0.29, respectively) (Fig. 4). Next, we performed these experiments in the older *Spr* ^−/−^ mice at the age of 4–8 months. The contraction of the aortic rings in response to high KCl from the older *Spr* ^−/−^ mice (2.57 ± 0.41 mN) showed an increasing trend compared with that in the wild‐type mice of the same age (1.69 ± 0.17 mN) (*P* = 0.065, *n* = 10, Student's *t*‐test). Although the contractile response to phenylephrine relative to high KCl was not significantly different between two genotypes (Fig. 5A), the vasodilatory response to ACh was significantly lower in the aortic rings from the older *Spr* ^−/−^ mice than those from wild‐type, as shown by the rightward shift of the dose–response curve (Fig. 5B). The relaxation response to ACh disappeared in the presence of l‐NAME in both genotypes, which proved that the response was caused by endothelium‐derived NO (Fig. 5C). There was no significant difference in the vasodilatory response to SNP in the presence of l‐NAME in either genotypes, which indicated that the direct relaxation ability of the vascular smooth muscle caused by an NO donor was not impaired in *Spr* ^−/−^ mice (Fig. 5D).

### Protein amounts of *α*_1_ adrenergic receptor and eNOS in the aorta were not significantly different between Spr^−/−^ mice and wild‐type animals

**Figure 6:**
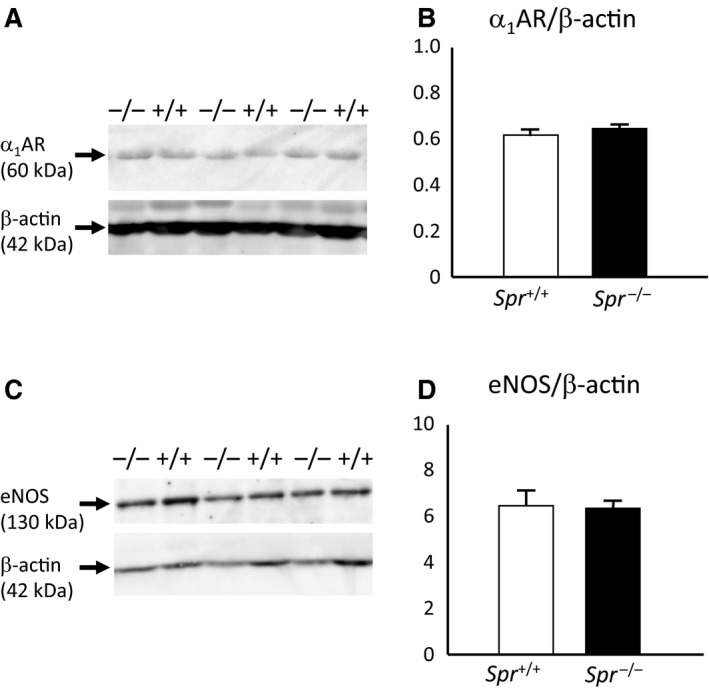
Protein amounts of *α*_1_ adrenergic receptor and eNOS in the aorta of wild‐type mice and *Spr* ^−/−^ mice. (A) Western blotting of *α*_1_ adrenergic receptor (*α*_1_ AR) and *β*‐actin. Upper panel; *α*_1_ adrenergic receptor, lower panel *β*‐actin. Genotypes are indicated on the top of the panel. (B) Quantification of the protein amount of *α*_1_ adrenergic receptor relative to that of *β*‐actin. Wild‐type mice; open bar, *Spr* ^−/−^ mice; closed bar. (C) Western blotting of eNOS and *β*‐actin. Upper panel; eNOS, lower panel *β*‐actin. Genotypes are indicated on the top of the panel. (B) Quantification of the protein amount of eNOS relative to that of *β*‐actin. Wild‐type mice; open bar, *Spr* ^−/−^ mice; closed bar. Differences in B and D are not significant (*n* = 6, Student's *t*‐test).

We then examined the expression of *α*_1_ adrenergic receptor and eNOS in the aorta of the older *Spr* ^*−/−*^ and wild‐type ones. Protein amount of *α*1 adrenergic receptor relative to *β*‐actin was not significantly different between *Spr* ^*−/−*^ (0.64 ± 0.02) and wild‐type mice (0.61 ± 0.02) (*P* = 0.26, *n* = 6, Student's *t*‐test) (Fig. 6A,B). Protein amount of eNOS relative to *β*‐actin was unaltered between *Spr* ^*−/−*^ (6.39 ± 0.38) and wild‐type mice (6.53 ± 0.65) (*P* = 0.86, *n* = 6, Student's *t*‐test) (Fig. 6C,D).

### Spr^−/−^ mice exhibited severe hypoglycemia and elevation of plasma renin activity

**Figure 7:**
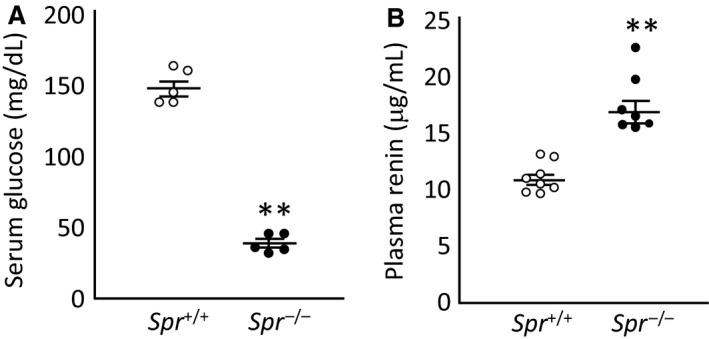
Serum glucose concentration and plasma renin activity of wild‐type mice and *Spr* ^−/−^ mice. (A) Serum glucose concentrations in wild‐type mice and *Spr* ^−/−^ mice after a 17‐h fast. The serum glucose level of *Spr* ^−/−^ mice was significantly lower than that of wild‐type animals. (B) The plasma renin activity in *Spr* ^−/−^ mice was 1.6 times higher than in their wild‐type littermates. Each circle represents one mouse sample. Wild‐type mice; open circles, *Spr* ^−/−^ mice; closed circles. The averages and SEMs are indicated by bars. Significant differences between the two genotypes are indicated with asterisks (**;*P* < 0.01, *n* = 5 in panel A, *n* = 7 and 8 in panel B, Student's *t*‐test).

We found that *Spr* ^−/−^ mice showed extreme hypoglycemia in the serum (38.0 ± 2.5 mg/dL) after 17 h of fasting, in contrast to that in wild‐type controls (145.6 ± 5.7 mg/dL) (Fig. 7A). We determined the SBP of mice of each genotype after 17 h of starvation and 1 h after feeding with pellets and compared the fluctuation in SBP before and after feeding. The difference was not significant; however, *Spr* ^−/−^ mice tended toward a decrease in SBP (−36.4 ± 21.5 mmHg), while the wild‐type animals had increased SBPs (12.6 ± 6.9 mmHg) (*P* = 0.056, *n* = 6, Student's *t*‐test). There was no significant difference in the fluctuation in the simultaneously recorded HR (*Spr* ^−/−^ mice; 58.3 ± 11.1 beats/min, wild‐type mice; 45.8 ± 11.5 beats/min, *P* = 0.45, *n* = 6). The plasma renin activity in the *Spr* ^−/−^ mice (17.3 ± 0.99 *μ*g/mL) was 1.6‐fold higher than that in the wild‐type mice (10.9 ± 0.47 *μ*g/mL) (Fig. 7B). The concentrations of nitrites and nitrates in the plasma of *Spr* ^−/−^ mice (31.5 ± 10.6 *μ*mol/L) were not significantly different from those in the wild‐type animals (21.4 ± 3.4 *μ*mol/L) (*P* = 0.39, *n* = 6, Student's *t*‐test).

## Discussion

BH4 biosynthesis was severely impaired by the disruption of the *Spr* gene in *Spr* ^−/−^ mice, which caused the depletion of catecholamines and serotonin in both the central and peripheral nervous systems and tissues. Thus, we sought to determine the mechanism underlying the hypertension with fluctuation observed in *Spr* ^−/−^ mice despite impaired monoamine biosynthesis.

First, as the norepinephrine contents in the sympathetic nerve terminals is reduced, the neurotransmitter would be consumed rapidly in accordance with sympathetic activity; however, compensatory pre‐ and/or postsynaptic hypersensitivity would occur (Teasell et al. 2000). HRV spectral analysis, which revealed that the LF/HF and LF/TF ratios measured in the *Spr* ^−/−^mice were significantly higher than those in the wild‐type mice, suggested that the sympathetic input far surpassed the parasympathetic input, regardless of monoamine deficiency. We consider that the imbalance of sympathetic and parasympathetic nervous system would contribute to cardiovascular instability of *Spr* ^−/−^ mice; however, it could not be explained by hypersensitivity of *α*_1_ adrenergic receptor in the aorta, for phenylephrine‐contraction relative to high KCl and protein amount of *α*_1_ adrenergic receptor in the aorta was not significantly altered. The changes in excitation–contraction coupling including Ca^2+^ sensitivity (Bentzer et al. 1997) possibly contributed because vasocontraction to high KCl tended to be exaggerated in the older *Spr* ^−/−^ mice. We do not deny involvement of centrally mediated dysregulation of the autonomic nervous system.

In addition, we found that *Spr* ^−/−^ mice suffered from hypoglycemia, which chronically stimulates the sympathetic nervous system and drives feeding behavior; then, a rapid increase in blood glucose by food‐intake and repletion of the gastrointestinal tract would cause a shift from the sympathetic dominant stage to the parasympathetic dominant stage. This would be one factor contributing to the fluctuation in blood pressure in *Spr* ^−/−^ mice. The cause of hypoglycemia is unknown; however, we hypothesized that catecholamine insufficiency and hyperphenylalaninemia (Yang et al. 2006; Homma et al. 2011) would impair glycogenolysis and glucogenesis. Furthermore, BH4 deficiency would reduce neuronal NOS (nNOS) activity, and the mice could not take in a sufficient amount of food, similar to the pyloric stenosis‐like symptoms reported in *nNOS* ^−/−^ mice (Huang et al. 1993).

The second mechanism underlying the unstable hypertension in *Spr* ^−/−^ mice is the loss of vascular flexibility caused by a reduction in endothelial NO. Although eNOS expression in the aorta of *Spr* ^−/−^ mice was not significantly changed and the *K*_*m*_ value of BH4 for NOS is extremely low (0.02–0.03 *μ*mol/L) (Thöny et al. 2000), severe depletion of BH4 in the endothelium would impede the vascular relaxation response. There are many articles indicating that BH4 deficiency or an imbalance in BH4 and BH2, its oxidized form, causes endothelial NOS (eNOS) dissociation and interferes with NO synthesis, resulting in radical production and vascular dysfunction (Klatt et al. 1995; Cai et al. 2002, 2005; Landmesser et al. 2003). This would explain the diastolic hypertension and elevation of plasma renin activity in *Spr* ^−/−^ mice. Renin would be secreted in response to the reduction in renal blood flow caused by endogenous vascular contraction and possibly exaggerated the hypertension in *Spr* ^−/−^ mice. Elevated plasma renin activity was also reported in mice lacking eNOS, which suffer from hypertension and bradycardia (Shesely et al. 1996).

As shown in Table 1 and 2, unexpectedly high amounts of BH4 or biopterin were detected in several tissues of *Spr* ^−/−^ mice, including the brain. It is possible that BH4 may be supplied via a minor alternative biosynthetic pathway (Hirakawa et al. 2009) in these tissues, and this may explain why the concentration of NO metabolites in the plasma of the *Spr* ^−/−^ mice was not significantly lower than that in the wild‐type animals. However, BH4 synthesized in other tissues cannot be easily uptaken by endothelial cells, because it was reported that enhanced vasoconstriction in response to phenylephrine and impaired vasodilation in response to ACh were observed in endothelial cell‐specific GTP cyclohydrolase 1 (GCH1) knockout mice (*GCH1* ^*fl/fl*^Tie2*Cre*) (Chuaiphichai et al. 2014). Furthermore, *GCH1* ^*fl/fl*^Tie2*Cre* mice showed a significant but relatively small increase in systolic blood pressure. We suggest that one of the major differences between *Spr* ^−/−^ and *GCH1* ^*fl/fl*^Tie2*Cre* mice is the contribution of BH4 and monoamine deficiency in the central nervous system.

One of the biological impacts of *Spr* ^−/−^ mice is the possible contribution of BH4 insufficiency to age‐related hypertension in human. The endothelium‐specific reduction in SPR in deoxycorticosterone acetate (DOCA)‐salt hypertensive mice suggests the importance of SPR in maintaining normal blood pressure (Youn et al. 2012). Yang et al. (2009) reported that eNOS uncoupling in the mesenteric artery and impairment of the vasodilatory response to ACh were exaggerated by aging in mice; these effects were rescued by supplementation of sepiapterin. Age‐related reductions in BH4 in rat skeletal muscle arterioles were reported (Delp et al. 2008; Pierce and LaRocca 2008). The level of pterin cofactors in the urine, serum (Shintaku et al. 1982), and cerebrospinal fluid (Williams et al. 1980) also tend to decrease with age in humans. We believe that the effect of steroids might be the other factor explaining these deteriorations in vascular response and the gender differences in DBP.

The monoaminergic nerve terminals are functionally impaired by the reduction in monoamine biosynthesis in BH4‐deficient mice, which was caused by decreased TH protein, as shown by our previous studies (Sumi‐Ichinose et al. 2001, 2005). We speculate that there might be similar mechanisms underlying the cardiovascular instability of patients with PD, MSA, and SCI as in the case of *Spr* ^−/−^ mice because of the following observations and reports. First, it was reported that *α*‐synuclein might affect the biosynthesis of BH4 (Baptista et al. 2003; Ryan et al. 2014); thus, norepinephrine biosynthesis may be impaired by a reduction in BH4 due to *α*‐synuclein accumulation in the sympathetic neurons in patients with PD and MSA, especially in the earlier stages (Braak et al. 2003). Second, the aortic relaxation response to ACh was significantly lower in transgenic mice expressing *α*‐synuclein in TH‐positive nerve fibers (Marrachelli et al. 2010). In some types of MSA, the central input to the peripheral nervous system is disrupted by the degeneration of central autonomic nuclei with glial inclusion bodies containing *α*‐synuclein (Fanciculli and Wenning 2015), and a transectional lesion of the spinal cord disrupts that in SCI. Patients with SCI often demonstrate a sharp elevation in blood pressure due to sudden vasoconstriction, which is easily triggered by afferent stimulation, such as an over‐full bladder, constipation, and mental stress; this effect is known as autonomic dysreflexia (Karlsson et al. 1998). Using rat models, Laird et al. (2008) indicated that a reduction in the norepinephrine transporter occurs in sympathetic nerve terminals, which increases the norepinephrine concentration in the synaptic cleft, causing vascular hypersensitivity. Further studies are needed to answer the remaining questions.

## Conflict of Interest

The authors declare that no conflict of interest exists.
